# PW02-035 - A role for thermo-TRP channels in innate immunity?

**DOI:** 10.1186/1546-0096-11-S1-A176

**Published:** 2013-11-08

**Authors:** M Stoffels, T Remijn, LM Elders, HD de Koning, JW van der Meer, A Simon

**Affiliations:** 1 Department of General Internal Medicine, Radboud University Nijmegen Medical Centre, Netherlands; 2 Nijmegen Institute for Infection, Inflammation and Immunity (N4i), Netherlands; 3 Nijmegen Centre for Molecular Life Sciences (NCMLS), Netherlands; 4 Department of Dermatology, Radboud University Nijmegen Medical Centre, Nijmegen, Netherlands

## Introduction

Exposure to cold can induce an exaggerated (local and systemic) inflammatory response in a number of rare disorders, including cryopyrin-associated periodic syndrome (CAPS), and idiopathic cold urticaria (CU). Although it is widely recognized that temperature sensing in neurons is mediated by several transient receptor potential (TRP) channels, it is not known how immune cells sense cold temperatures.

## Objectives

In the present study we aimed to explore how inflammatory cells sense cold.

## Methods

qRT-PCR, western blot and immunohistochemistry were used to detect TRP mRNA and protein in several human-derived cell lines, primary cells and skin biopsies. Cytokine concentrations in culture supernatants of stimulation assays were detected by ELISA.

## Results

mRNA of different thermo-TRPs was detected in PBMCs, macrophages and keratinocytes. The ‘cool’ menthol receptor TRPM8 is differentially expressed in glycosylated form in immune cells, human fibroblast and lymphoblast cell lines. TRPM8 expression was detected in skin biopsies and localized to the keratinocytes and epithelial cells lining blood vessels. No differences in expression were observed between biopsies from healthy controls and CAPS or CU patients. Preincubation of PBMCs with menthol, a TRPM8-agonist, resulted in enhanced interleukin-1 beta (IL-1β) secretion in response to TLR stimulation.

## Conclusion

TRPM8 is differentially expressed in human immune cells in glycosylated form, indicating active regulation. *Ex vivo* stimulation of PBMCs with menthol results in an increased inflammatory response to TLR stimuli. We hypothesize that *in vivo* cold exposure results in a modulated inflammatory response, through activation of temperature sensitive ion channels. This activation is most likely regulated at the post-translational level.

## Competing interests

None declared

